# Correlational and Molecular Mechanism Analyses of Bioactive Compounds From *Ziziphus jujuba*: Focusing on Anti‐Hyaluronidase and Antioxidant Networks

**DOI:** 10.1002/fsn3.71844

**Published:** 2026-05-07

**Authors:** Saba Noreen, Zhaoyang Wu, Sichan Kim, Ji Won Choi, Soo Kyeong Lee, Deokho Lee, Soon Sung Lim

**Affiliations:** ^1^ Department of Food Science and Nutrition Hallym University Chuncheon Republic of Korea; ^2^ Korean Institute of Nutrition Hallym University Chuncheon Republic of Korea

**Keywords:** antioxidants, hyaluronic acid, hyaluronidase, ultrafiltration‐HPLC, *Ziziphus jujuba*

## Abstract

Skin aging, impairments in skin hydration and elasticity, are associated with loss of hyaluronic acid (HA) and an increase in oxidative stress. As hyaluronidase mainly contributes to HA degradation and disruption to the extracellular matrix (ECM), it has been considered a potential method of preventing skin aging. In this study, we aimed to examine phytochemical compositions and multi‐target enzyme inhibitory capacities of *Ziziphus jujuba* extracts from South Korea, emphasizing antioxidant and hyaluronidase inhibiting activities. Using ultrafiltration high‐performance liquid chromatography (UF‐HPLC), electron ionization mass spectrometry (EI‐MS), and NMR techniques, we identified jujuboside B (1), p‐coumaric acid (2), medicagenic acid (3), rutin (4), and luteolin 7‐O‐Glucoside (5) as key bioactive constituents. Medicagenic acid (IC_50_ = 113 μM) showed the strongest predicted interaction with hyaluronidase with a high docking score. Hyaluronidase inhibition in *Z. jujuba* is primarily mediated by a distinct subset of triterpenoid and phenolic compounds having antioxidant activities. Based on our data, *Z. jujuba* extract may be a promising natural source of bioactive compounds that could be relevant for skin health due to their ability to modulate enzymes, especially antioxidants and anti‐hyaluronidase. Collectively, these findings suggest that *Z. jujuba* derived compounds have potential as candidates for further investigation in dermatological and functional food applications.

## Graphical abstract

Extract of *Z. jujuba* leaves and branches is prepared by using ethanol. The samples are examined for antioxidant and various enzyme inhibition activities. Then, bioactive components are isolated by column chromatography and identified by EI‐MS, NMR, and UV‐HPLC. Medicagenic acid may be the best candidate for anti‐hyaluronidase.

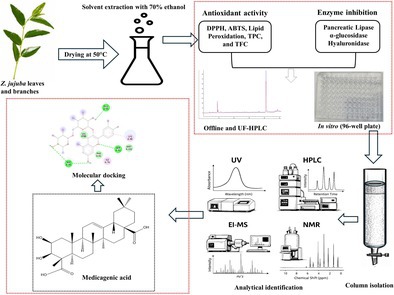

## 1 Introduction

Skin health concerns, such as premature aging, dryness, and loss of elasticity, have increased worldwide, particularly among adults over the age of 40 (Chylińska and Maciejczyk 2025; Chmielewski and Lesiak 2024). When people age, hyaluronic acid (HA) production decreases, leading to a reduction in water‐binding capacity, increases in drier and less elastic skin, and the formation of wrinkles (Chylińska and Maciejczyk 2025; Yoshida et al. 2018). HA, renowned for its anti‐inflammatory and antioxidant properties, may have pivotal roles in modulating reactive oxygen species (ROS) levels against oxidative damage (Zhou and Yu 2025; Xu et al. 2022; Zhang et al. 2022). According to a meta‐analysis, HA treatment has been found to improve skin hydration and radiance (Zhou and Yu 2025), implying that HA is required for anti‐skin aging.

One of the main contributors for the HA depletion is a hyaluronidase breaking down HA polymers within the extracellular matrix (Younis et al. 2022; Liu et al. 2023; Stern and Jedrzejas 2006; Lu et al. 2025). Excessive activities of hyaluronidase accelerate HA loss, resulting in drier, less resilient skin and the formation of wrinkles (Kang et al. 2021). However, skin health is not only influenced by HA degradation; it is also affected by other metabolic processes, including oxidative stress (Zhang et al. 2022; Younis et al. 2022; Liu et al. 2023; Stern and Jedrzejas 2006).

*Ziziphus jujuba (Z. jujuba)* is a native plant in East Asia and widely cultivated in South Korea (Cho et al. 2026). *Z. jujuba* is recognized as a rich source of polyphenols, flavonoids, triterpenes, and polysaccharides with diverse biological activities. Previous studies (Li et al. 2017) showed that this plant has antioxidant (Singh et al. 2022), anti‐inflammatory (Kandimalla et al. 2016; Yu et al. 2012), hepato‐protective, and several metabolic enzyme inhibitory effects, including inhibition of digestive enzymes such as α‐glucosidase (Contrast Agent 2019) and pancreatic lipase (PL) (Gou et al. 2023; Kharyal and Puri 2025; Rybak and Wojdyło 2023). Despite extensive research documenting the antioxidant and metabolic enzyme inhibitory activities of *Z. jujuba* , its effects on skin‐related enzymes such as hyaluronidase remain largely unexplored.

In this study, we aimed to analyze the phytochemical constituents of *Z. jujuba* extracts, emphasizing concentration‐dependent hyaluronidase inhibition mediated by specific constituents identified via ultrafiltration paired with high‐performance liquid chromatography (UF‐HPLC). Furthermore, we identified the most powerful hyaluronidase inhibitor in *Z*. *jujuba* extracts. Based on our data, *Z. jujuba* extract has potential as a natural source for anti‐skin aging by regulating metabolic enzymes, especially antioxidants and anti‐hyaluronidase.

## 2 Materials and Methods

### 2.1 Chemicals and Reagents

2,2′‐Azobis (2‐amidinopropane) dihydrochloride (AAPH), thio barbituric acid (TBA), trichloroacetic acid (TCA), linoleic acid, Tween 20, iron sulfate (FeSO_4_), folin–ciocalteu, sodium carbonate (Na_2_CO_3_), sodium nitrite (NaNO_2_), sodium hydroxide (NaOH), α‐glucosidase enzyme, PNPG‐4‐Nitrophenly‐B‐D‐glucopyranoside, type‐1‐S bovine testes hyaluronidase, sodium hyaluronate, calcium chloride (CaCl_2_), sodium tetraborate (Na_2_B_4_O_7_), ρ‐dimethyl amino benzaldehyde, PL enzyme, p‐nitrophenol (pNP), acarbose, tanic acid, trolox, gallic acid, orlistat, catechin, and Amicon Ultra‐0.5 Centrifugal Filter Unit with a 10 kDa MW cut‐off membrane (Millipore, Bedford, MA, US) were purchased from Sigma‐Aldrich Chemical Co. (St. Louis, MO, USA). Methanol and dimethyl sulfoxide (DMSO) were purchased from J. T. Baker Co. (Phillips‐burg, NJ, USA) for sample preparation and analysis. Ultrapure water used in this study was produced using a Milli‐Q water purification system (Millipore Co., Bedford, MA, USA). The whole parts of *Z. jujuba* were collected from South Korea in 2025. The dried samples and separated compounds were placed at the Center for Efficacy Assessment and Development of Functional Foods and Drugs at Hallym University, Chuncheon, Gangwon, South Korea.

### 2.2 Preparation of *Z. jujuba* Extract

The whole aerial parts (leaves and branches) of *Z. jujuba* were obtained from Chuncheon, Gangwon, Republic of Korea (37.88° N, 127.73° E). Voucher specimen (No. KIN‐P825) was authenticated by Prof. Soon Sung Lim and Dr. Deokho Lee.

From the whole part, a mixture of the dried *Z. jujuba* leaves and branches (100 g) was extracted using 70% ethanol at 35°C for 72 h. The extract was filtered through filter papers and evaporated with a rotary evaporator (Heidolph Hei‐VAP Rotary Evaporator) under reduced pressure at 37°C. The dried extract was weighed to calculate the extraction yield: 18.3% (w/w) based on the dry weight of the plant material. The resulting extracts were kept at 4°C until they were analyzed.

### 2.3 Enzyme Inhibition and Antioxidants Assays

#### 2.3.1 Lipid Peroxidation

Lipid peroxidation was assessed with minor modifications, as described by Okumus et al. and Shin et al. (Okumus 2024; Shin et al. 2001). Briefly, each sample extract at the concentrations of 1, 0.5, and 0.1 mg/mL in methanol was added to a linoleic acid‐Tween 20 emulsion prepared in phosphate‐buffered saline (PBS, pH 7.4). Subsequently, FeSO_4_ solution (20 mM in PBS, pH 7.4) was added to initiate the solution. The reaction mixtures were incubated in the dark room at 37°C for 16 h. After incubation, the reaction mixture was transferred to a new tube and mixed with 0.67% TCA. Then, 20% TBA solution was added to the supernatant. The resulting mixture was heated in a boiling water bath at 95°C for 30 min to allow the formation of the TBA‐malondialdehyde adduct. After cooling, the samples were centrifuged to remove any insoluble particles. The absorbance of the supernatant was then measured at an appropriate wavelength, 532 nm, to determine the extent of lipid peroxidation by using an EL‐800 Universal microplate reader (Bio‐Tek Instrument Inc., Winooski, USA). Trolox was used as a positive control. The results were expressed as the IC_50_ of lipid peroxidation.

#### 2.3.2 Total Phenolic Content (TPC)

The TPC of *Z. jujuba* was determined by a method from previous literature (Paganotti and Barbeira 2024) with some modifications: the Folin–Ciocalteu colorimetric method. Briefly, gallic acid or the sample extract in methanol solution at different concentrations was added to a tube, and the volume was made up to 10 mL using distilled water. After adding Folin–Ciocalteu reagent and sodium carbonate solution (20%, w/v), the tube was placed in a boiling water bath for 1 min and cooled at constant volume to 25 mL. The mixture was then blended and kept in the dark room at room temperature for 30 min. The absorbance of the resulting blue color was measured at 745 nm using an EL‐800 Universal microplate reader (Bio‐Tek Instrument Inc., Winooski, USA) against a blank containing 0.2 mL of methanol. TPC was expressed as milligrams of gallic acid equivalents per gram of extract (mg GAE/g extract).

#### 2.3.3 Total Flavonoid Content (TFC)

The TFC of *Z. jujuba* was determined by a method from previous literature (Jha et al. 2024) with some modifications. An aliquot of the standard solution of catechin at different concentrations and/or our samples were added to volumetric flasks containing distilled water. At the onset of the experiment, 5% NaNO_2_ was added to the flask. After 5 min, 10% AlCl_3_ was added. Then, 1 M NaOH was added to the mixture. Immediately, the solution was diluted to a final volume of 10 mL with distilled water and mixed thoroughly. Absorbance of the mixture was determined at 510 nm versus the prepared blanks. TFC was expressed as milligrams of quercetin equivalents per gram of extract (mg QE/g extract).

#### 2.3.4 Pancreatic Lipase (PL)

The PL inhibitory activity of the extract was determined using the method of Zhang et al. (2020), with minor modifications. The substrate pNPB is hydrolyzed by the PL enzyme to p‐nitrophenol (pNP). Assay buffer (13 mM Tris–HCl, pH 8.0, 1.3 mM CaCl_2_, and 150 mM NaCl) was used to dissolve PL with a 1 mg/mL dilution rate. The PL supernatant was collected after centrifuging at 3200×*g* for 15 min at 25°C. The mixture of plant sample (1, 0.5, 0.1 mg/mL dissolved in 50% methanol) and enzyme (1 mg/mL) was put into 96‐well microliter plates and incubated for 10 min at 37°C before use. The enzyme reaction at 37°C is started with the addition of pNPB (2 mg/mL in assay buffer). Then, the reaction was observed at 405 nm. An EL800 Universal microplate reader (Bio‐Tek Instrument Inc., Winooski, USA) was used to record the absorbance at 405 nm. The results were also expressed as IC_50_ of the pancreatic lipase inhibition.

#### 2.3.5 α‐Glucosidase

The α‐glucosidase inhibitory activity was evaluated according to a previous study from Flores‐Daou et al. (Daou et al. 2022), with some modifications. In brief, α‐glucosidase solution (0.015 mg/mL in PBS buffer pH 7.0) was mixed with phosphate buffer (pH 7.0) and sample solution at the concentrations of 1, 0.5, and 0.1 mg/mL in methanol. The mixture was incubated at 37°C for 5 min, and then PNPG‐4‐Nitrophenly‐B‐D‐ glucopyranoside solution (1 mM in PBS buffer pH 7.0) was added to the mixture. Then the mixture was put for reaction at 25°C for 30 min. The absorbance of the reaction mixture was determined at 405 nm using a microplate reader EL‐800 Universal microplate reader (Bio‐Tek Instrument Inc., Winooski, USA). Acarbose was used as the positive control. The results were also expressed as IC_50_ of the α‐glucosidase inhibition.

#### 2.3.6 Hyaluronidase

Hyaluronidase inhibitory activity was measured as previously described by Okada et al. (2025) with a few modifications. Type‐1‐S bovine testes hyaluronidase dissolved in 0.1 M acetate buffer (pH 3.5) was mixed with extract (1, 0.5, 0.1 mg/mL) dissolved in 50% methanol and incubated in a water bath at 37°C for 20 min. 12.5 mM calcium chloride was added to the reaction mixture, and then the mixture was incubated at 37°C for 10 min. This Ca2^+^ activated hyaluronidase was treated with sodium hyaluronate (12 mg/mL) dissolved in 0.1 M acetate buffer (pH 3.5), and then incubated in a water bath at 37°C for 40 min. 0.9 M sodium hydroxide and 0.2 M sodium tetraborate were added to the reaction mixture, and then incubated in a boiling water bath for 3 min. After cooling to room temperature, ρ‐dimethyl amino benzaldehyde solution dissolved in 100% acetic acid and 10 N hydrochloric acid was added to the reaction mixture, which was then incubated in a water bath at 37°C for 10 min. The control group was treated with 5% DMSO instead of the extract. The absorbance of the reaction mixture was determined at 585 nm using a microplate reader EL‐800 Universal microplate reader (Bio‐Tek Instrument Inc., Winooski, USA). The IC_50_ values were determined by nonlinear regression of the dose–response curves using OriginPro 2024 (OriginLab Corp., Northampton, MA, USA). All assays were performed in triplicate at three concentrations (1, 0.5, and 0.1 mg/mL) and results are expressed as mean ± SD (*n* = 3 independent experiments).

### 2.4 Offline Antioxidants and Ultrafiltration (UF)‐HPLC Binding Assay

#### 2.4.1 HPLC Analysis

The sample extracts were analyzed to compare using equipment from Agilent Technologies (Santa Clara, CA, USA). The setup consisted of a G1311A pump, a G1329A automated sample injector, a G1316A column oven kept at 30°C, and a G1314D detector. The HPLC mobile phases used were acidic water 0.1% formic acid (A) and acetonitrile (B). The sample was analyzed at 254 nm and separated with a flow rate of 0.5 mL/min using a PAK‐C18 column (250 × 4.6 mm, 5 μm). The separation process was as follows: 10% B from 0 to 5 min, 10%–20% B from 5 to 40 min, 20%–100% B from 40 to 55 min, and 100% B from 60 to 75 min. The fractions were also analyzed at 254 nm and separated with a flow rate of 0.5 mL/min using the same method (Table S1).

Before injection, the *Z. jujuba* extract and the isolated compounds were dissolved in HPLC‐grade methanol and filtered through a 0.22 μm PTFE syringe filter. The final concentrations were 1.0 mg/mL for the crude extract and 0.1–0.5 mg/mL for the individual compounds for analytical HPLC. The injection volume was 10 μL for all chromatographic runs, including the offline AAPH and UF HPLC assays.

#### 2.4.2 2,2′‐Azobis (2‐Amidinopropane) Dihydrochloride (AAPH) Offline

The AAPH offline HPLC activity of the extract was determined using the method of Wu et al. (2023). Briefly, a mixture of AAPH solution (16 mmol in PBS with a pH of 7.4) and the sample (1 mg/mL in methanol) was incubated for 30 min at 80°C. Therefore, the reaction solution with an injection volume of 10 μL was analyzed using HPLC. As a blank, the AAPH solution was replaced with PBS when incubating with the extract to form an AAPH‐free group. Compared to the AAPH‐free group, compounds with reduced peak areas in the AAPH group were assigned as having a potential antioxidant activity. In addition, to study the effect of heating temperature on polyphenols, we set up a group, a mixture of PBS and the extract without heating, as a control group. Peak area reduction by heating and AAPH radicals was calculated as the following formulas and, respectively:

$$\text{Peak reduction}\;\mathrm{by}\;\text{heating}=\left(A_{\text{control}}-A_{\text{AAPH free group}}\right)/A_{\text{control}}\times 100\%$$

$$\begin{array}{c} \text{Peak reduction}\;\mathrm{by}\;\text{AAPH radicals}\\ =\left(A_{\text{AAPH free group}}-A_{\text{AAPH group}}\right)/A_{\text{AAPH free group}}\times 100\% \end{array}$$

#### 2.4.3 Ultrafiltration (UF)‐Pancreatic Lipase (PL), UF‐α‐Glucosidase, UF‐Hyaluronidase

The UF‐PL and UF‐α‐glucosidase and hyaluronidase inhibitory activities of the extract were determined using methods from Wei et al. (2025), Li, He, et al. (2021), and Zhou et al. (2023) with minor modifications. The proposed strategy was performed using three steps: co‐incubation, UF, and HPLC identification.

Briefly, the extract solution (30 μL, 2 mg/mL) was mixed with enzyme solution (180 μL, 1500 U/mL prepared in the appropriate buffer), giving a sample‐to‐enzyme ratio of 1:6 (v/v). The mixture was incubated at 37°C for 30 min to allow enzyme ligand interaction. The incubation mixture was then transferred to an Amicon Ultra‐0.5 centrifugal filter unit with a 10 kDa molecular weight cutoff membrane. A 10 kDa ultrafiltration membrane was used to separate enzyme–ligand complexes from unbound small molecules. The cutoff was selected considering the molecular weight of enzymes (~55–60 kDa), allowing the enzyme and its bound ligands to be retained during ultrafiltration (Millipore, Bedford, MA, USA) and centrifuged at 11,000 rpm for 30 min at 4°C. The retained enzyme ligand complexes were washed twice with 200 μL of buffer solution to remove unbound compounds. After ultrafiltration, the filtrates were subjected to HPLC analysis to identify potential enzyme‐binding ligands. The binding capacities of constituent enzymes were determined by comparing the decreased peak area in HPLC chromatograms: Peak reduction % = non‐active peak area‐active enzyme peak area/non‐active peak area × 100%.

### 2.5 Isolation and Identification of Compounds

The major components of *Z. jujuba* 70% ethanol extract were isolated by column chromatography. Briefly, the 70% ethanol extract of *Z. jujuba* . (2 g) was dissolved in 20% methanol. Sephadex LH‐20 column (2 cm × 90 cm, Uppsala, Sweden) was loaded with 20% methanol (Guillen Quispe et al. 2017) before sample injection. Then, 20, 40, 60, 80, and 100% methanol (v/v) were sequentially eluted at a flow rate of 2.0 mL/min, yielding five major fractions (Křenek et al. 2014). Each fraction was collected in 40 mL portions and combined based on similar HPLC peaks (Li et al. 2017).

After vacuum evaporation, fractions 1–5 yielded a jujuboside B‐enriched fraction (0.135%, 135 mg) (Kim and Ko 2025), p‐coumaric acid (0.022%, 22 mg) (Bao et al. 2021), luteolin 7‐O‐glucoside (0.04%, 40 mg) (Yahia et al. 2020), rutin (0.056%, 56 mg) (Bao et al. 2021), and medicagenic acid (0.028%, 28 mg) (Bharati et al. 2025). The jujuboside B (Kim and Ko 2025) enriched fraction was further purified by reverse‐phase chromatography using a C18 column (2 cm × 90 cm, Uppsala, Sweden), eluting using methanol at a flow rate of 2.0 mL/min with solvent systems 20, 60, 80, and 100 (v/v) at a flow rate of 2.0 mL/min to obtain several subfractions (1–5). Jujuboside B fractions 0.025% (25 mg) were yielded after evaporation. Subfractions 1–5 showed a single symmetric peak corresponding to the retention time of all fractions (Li et al. 2017; Wu et al. 2013).

The identity of the detected compounds was initially evaluated by comparing their UV absorption spectra with those of the authentic reference compounds and was analyzed under identical conditions. The purity of each compound was evaluated through analytical HPLC testing at 254 nm using the same gradient method, which required a purity level of 95% for peak area assessment to be considered acceptable. Structural confirmation and the absence of detectable impurities were further verified by examining the EI–MS and ^1^H NMR spectra (Küpcü and Tokatlı 2025).

EI‐MS was performed after dissolving each sample in HPLC‐grade methanol (approximately 0.1–0.5 mg/mL), and high‐resolution mass spectra were recorded under standard conditions (ionization energy 70 eV, source temperature 200°C–250°C) on a JEOL JMS‐T2000GC mass spectrometer (JEOL Ltd., Tokyo, Japan) equipped with an electron ionization source, which allows for the observation of clear molecular ions and characteristic fragment ions consistent with previously reported mass spectrometric data of *Z. jujuba* constituents (Zhang et al. 2014).

NMR was carried out on a Bruker AV600 MHz FT‐NMR spectrometer (Bruker BioSpin, Rheinstetten, Germany); each compound was dissolved in DMSO‐d₆ or methanol‐d_4_, and ^1^H‐NMR spectra (600 MHz) were acquired at 25°C using standard pulse sequences, with chemical shifts (δ) referenced to residual solvent signals (Bozicevic et al. 2017).

### 2.6 Molecule Docking

Protein–ligand docking was carried out using the CB‐Dock2 web server (Cao Lab, http://cadd.zju.edu.cn/cb‐dock2), which performs cavity‐detection–guided blind docking based on AutoDock Vina. The three‐dimensional structure of hyaluronidase (PDB ID: 2PE4) obtained from the protein data bank (PDB) was uploaded after removal of water molecules and co‐crystallized ligands, and polar hydrogens and Gasteiger charges were assigned automatically by the server. Two‐dimensional structures of jujuboside B, p‐coumaric acid, medicagenic acid, rutin, luteolin 7‐O‐glucoside, and tannic acid were obtained from PubChem, energy‐minimized using ChemDraw/Chem3D (PerkinElmer). CB‐Dock2 automatically identified potential binding cavities and performed docking using AutoDock Vina scoring functions, generating multiple binding poses for each ligand. The best pose was selected based on the lowest binding energy. To provide complete validation, tannic acid, a known hyaluronidase inhibitor, was included as a reference ligand and docked under identical conditions.

In CB‐Dock2, the docking search space was defined automatically based on the predicted cavity coordinates and size, rather than being defined manually using a grid box. Docking was performed using the server's default AutoDock Vina settings, and the top‐ranked binding poses generated were inspected for interaction analysis.

### 2.7 Statistical Analysis

All experiments were performed in triplicate, and the results are presented as the mean ± standard deviation (SD). IC_50_ values were calculated using Microsoft Excel (Microsoft Corp., USA) via nonlinear regression analysis of the dose–response curves. Pearson's correlation coefficients were calculated by using OriginPro 2024 (OriginLab Corp., Northampton, MA, USA) to evaluate the relationships between TPC, TFC, lipid peroxidation inhibition, PL inhibition, α‐glucosidase inhibition, and hyaluronidase inhibition activities. The correlation matrix was visualized as a heatmap and a chord diagram. One‐way analysis of variance (ANOVA) was used to evaluate statistical significance, followed by a Tukey post hoc test. Differences were considered statistically significant at *p* < 0.05.

## 3 Result and Discussion

### 3.1 Lipid Peroxidation and Enzyme Inhibition Curves

**FIGURE 1:**
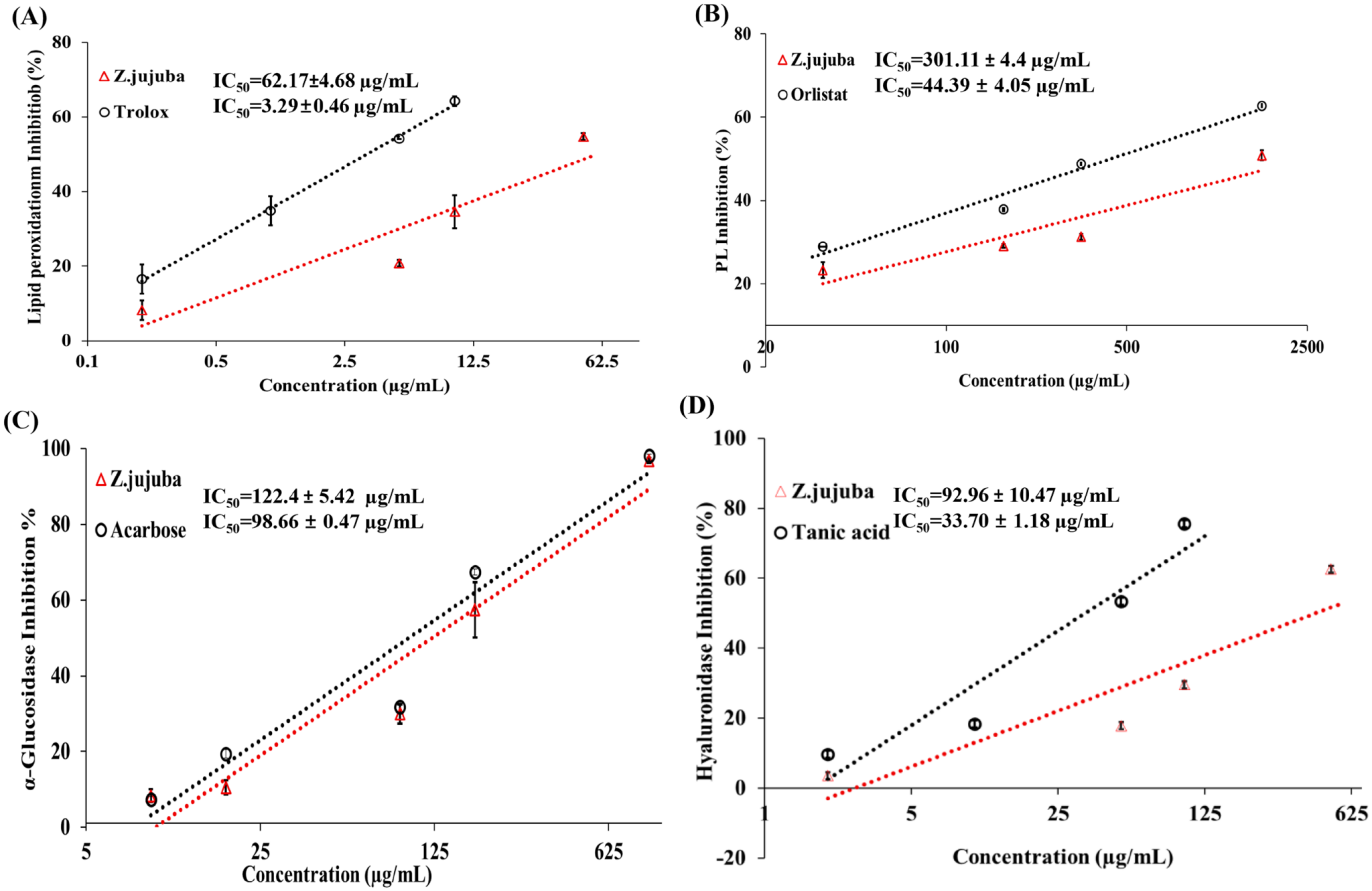
The inhibition rate by *Z. jujuba* of the lipid peroxidation and enzyme activity plots. (A) the inhibition curve of lipid peroxidation profile, (B) the inhibition curve of pancreatic lipase (PL) enzyme, (C) the inhibition curve of α‐glucosidase enzyme, and (D) the inhibition curve of hyaluronidase enzyme. Data are presented as mean ± SD (*n* = 3 independent experiments). IC_50_ values were calculated by non‐linear regression of concentration response curves.

The dose‐dependent inhibition profiles of *Z. jujuba* extract (Figure 1). The extract clearly exhibited concentration‐dependent inhibitory effects on lipid peroxidation and all three enzymes, with IC_50_ values of 62.17 ± 4.68 μg/mL for lipid peroxidation, 301.11 ± 4.40 μg/mL for PL, α‐glucosidase 122.40 ± 5.42 μg/mL, and hyaluronidase 92.96 ± 10.47 μg/mL. Although weaker than their respective positive controls (Trolox, Orlistat, Acarbose, and Tannic acid), these values still indicated biologically meaningful activities. These results are consistent with previous studies on *Z. jujuba* species, suggesting that *Z. jujuba* species have antioxidant effects and various enzymatic inhibiting activities (Rybak and Wojdyło 2023; Trifonova et al. 2021).

### 3.2 HPLC Elution and Offline AAPH Radicals Profiling

**FIGURE 2:**
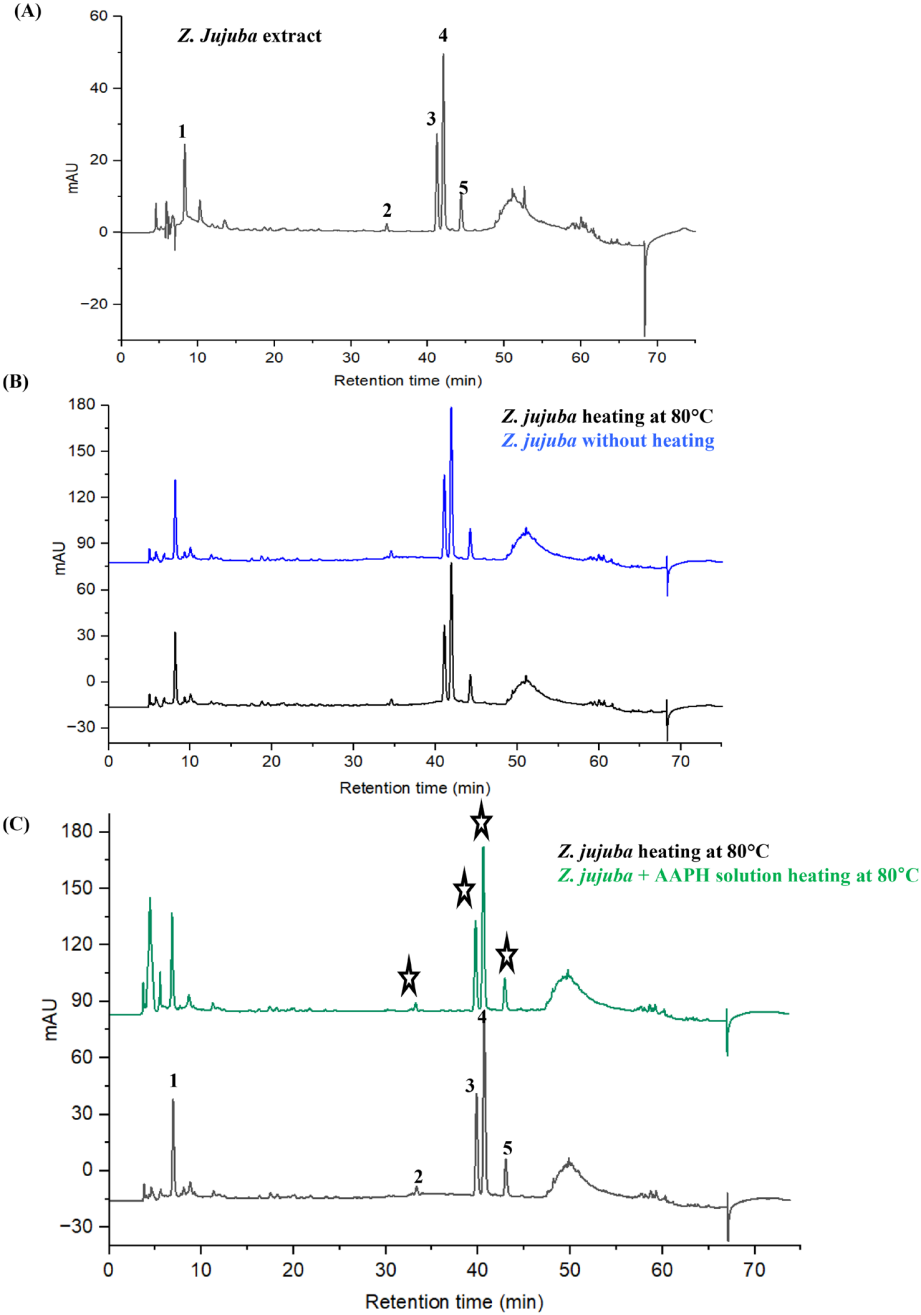
Antioxidant effects of *Z. jujuba* in Offline HPLC. (A) The HPLC elution pattern of *Z. jujuba*; (B, C) result of screening AAPH radicals; blue, green, and black peak lines represent numbers in the figure; stars represent a peak reduction of the compound based on a reaction with radicals.

The original chromatogram of *Z. jujuba* extract (Figure 2A) showed five major peaks (1–5). In offline AAPH‐HPLC analysis, the extract was heated at the same temperature with and without AAPH (80°C) to confirm that simple heating did not decompose these compounds (Figure 2B). However, when exposed to AAPH radicals, the intensity of specific peaks decreased selectively, suggesting these compounds were consumed during radical scavenging reactions (Figure 2C). As summarized in Table 1, peaks 3 and 4, identified as medicagenic acid and rutin, exhibited the largest peak area reductions (21.40% and 29.10%, respectively) and showed relatively low AAPH IC_50_ values (197 ± 3.40 and 127 ± 4.47 μM). In contrast, jujuboside B, p‐coumaric acid, and luteolin‐7‐O‐glucoside showed only negligible peak area reductions in this analysis, and no measurable IC_50_ values were observed. Our AAPH data is similar to previous reports' data of *Z. jujuba*, contributing to the inhibition peak area reduction of radicals and lipid peroxidation (Fu et al. 2021).

**TABLE 1:** Peak area reduction (%) of offline AAPH and enzymes by UF‐HPLC and IC_50_ μM values of isolated compounds.

Peak no.	Retention time (min)	Compound names	AAPH		PL		α‐Glucosidase		Hyaluronidase	
			Peak area reduction %	IC_50_ μM	Peak area reduction %	IC_50_ μM	Peak area reduction %	IC_50_ μM	Peak area reduction %	IC_50_ μM
1	8.101	Jujuboside B	—	—	—	—	11.65	—	28.49	179 ± 4.71
2	34.606	p‐Coumaric acid	5.78	—	—	—	—	—	20.06	205 ± 4.68
3	41.084	Medicagenic acid	21.40	197 ± 3.40	12.23	328 ± 6.76	24.58	177 ± 2.82	36.39	113 ± 1.99
4	41.931	Rutin	29.10	127 ± 4.47	20.78	127 ± 3.71	23.49	266 ± 1.92	30.14	186 ± 3.51
5	44.283	Luteolin 7‐O‐Glucoside	8.60	—	10.28	338 ± 3.82	11.62	—	19.52	211 ± 0.25

*Note:* AAPH, 2,2′‐Azobis(2‐amidinopropane) dihydrochloride. The IC_50_ μM values represent the concentration of each compound that inhibits the enzyme and AAPH radical activity by 50%. (−) The IC_50_ values could not be determined because the tested concentration range does not achieve 50% inhibition. (−) peak reduction in UF‐HPLC is below 10% threshold. Data are expressed as mean ± SD (*n* = 3), and error bars represent SD.

### 3.3 UF Binding Assays

**FIGURE 3:**
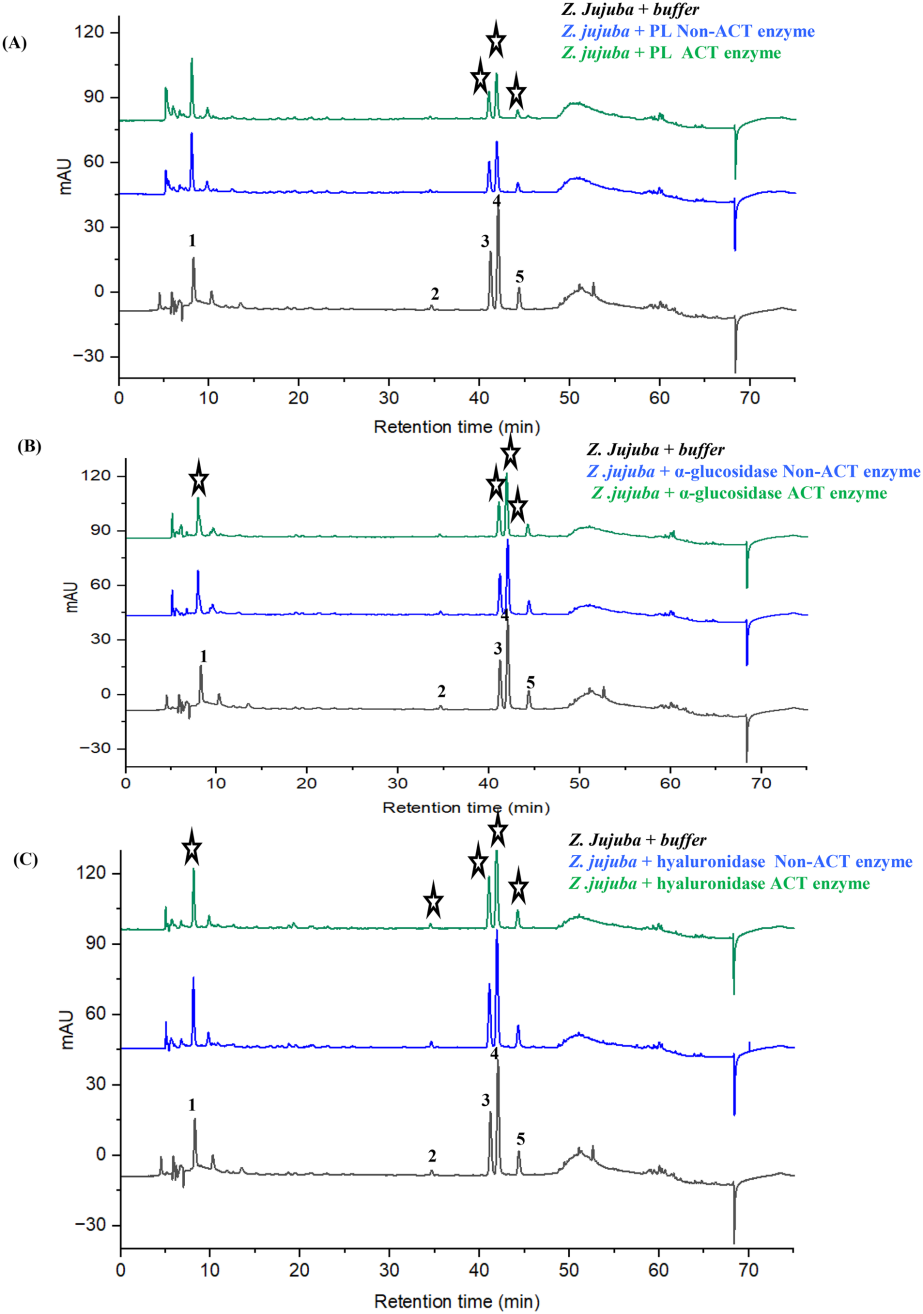
The peak area reduction rate by *Z. jujuba* of the enzymes by UF‐HPLC (A) result of comparison control, non‐active enzyme with active enzyme UF‐pancreatic lipase peak reduction, (B) result of comparison control, non‐active enzyme with active enzyme UF α‐glucosidase peak reduction, (C) result of comparison control, non‐active enzyme with active enzyme UF‐hyaluronidase peak reduction. Black lines represent the HPLC fraction of Control, blue lines represent the HPLC fraction with inactivated enzyme, and green lines represent the HPLC fraction with activated enzyme, and stars are showing peak reduction. *Control‐ *Z. jujuba* + buffer.

By applying UF‐HPLC (Huang et al. 2023) and comparing chromatograms obtained after incubation with active enzymes and heat‐inactivated enzymes, we tried to identify which components of *Z. jujuba* directly interact with each enzyme (Figure 3). In addition, a control (extract + buffer without enzyme) was included to evaluate potential non‐specific adsorption to the ultrafiltration membrane. The chromatographic profile of this control was similar to that of the non‐active enzyme sample, indicating that the observed peak reductions mainly result from ligand‐enzyme interactions rather than membrane binding artifacts. For PL (Figure 3A), the active enzyme chromatogram showed a significant decrease in peaks 3, 4, and 5 compared to the inactive control. Table 1 shows peak area reduction rates of 12.23%, 20.78%, and 10.28%, respectively, with corresponding IC_50_ values of 328 ± 6.76, 127 ± 3.71, and 338 ± 3.82 μM for medicagenic acid, rutin, and luteolin‐7‐O‐glucoside. This suggests that rutin may interact strongly with pancreatic lipase, which could explain the observed inhibitory activity. In the α‐glucosidase assay, the active enzyme caused significant reductions in peaks 1, 3, 4, and 5, with peak area reduction rates of 11.65% (jujuboside B), 24.58% (medicagenic acid), 23.49% (rutin), and 11.62% (luteolin‐7‐O‐glucoside), respectively (Figure 3B, Table 1). The corresponding IC_50_ values were 177 ± 2.82 μM for medicagenic acid and 266 ± 1.92 μM for rutin. However, since jujuboside B and luteolin‐7‐O‐glucoside failed to achieve a 50% inhibition rate within the tested range, these results suggest that medicagenic acid and rutin could be significant contributors to the extract's α‐glucosidase inhibitory activity.

In the hyaluronidase assay, the peak area decreased in all 1–5 peaks when treated with the active enzyme (Figure 3C). The peak area reduction rates (Table 1) were 28.49% (jujuboside B), 20.06% (p‐coumaric acid), 36.39% (medicagenic acid), 30.14% (rutin), and 19.52% (luteolin‐7‐O‐glucoside). The IC_50_ values were 179 ± 4.71, 205 ± 4.68, 113 ± 1.99, 186 ± 3.51, and 211 ± 0.25 μM, respectively. The inhibitory potency of medicagenic acid (IC_50_ = 113 μM) is comparable to that of several other natural hyaluronidase inhibitors, which have been reported in previous studies (Tables S2 and S3). Glycyrrhizin (IC_50_ = 3 μM) (Furuya et al. 1997) rosmarinic acid (IC_50_ = 240 μM) (Ippoushi et al. 2000) chicoric acid (IC_50_ = 171 μM) (Lengers et al. 2020) and nuciferin (IC_50_ = 240 μM) (Morikawa et al. 2019) have been shown to inhibit hyaluronidase. This indicates that plant‐derived polyphenols and triterpenoids can interact with the catalytic site of the enzyme and thereby modulate hyaluronic acid degradation (Li, Liu, et al. 2021). The IC_50_ value of medicagenic acid (113 μM, approximately 57 μg/mL) corresponds to a concentration that may be achievable in topical formulations containing approximately 0.4%–0.5% *Z. jujuba* extract, assuming the measured compound. Among the compounds identified in the present study, medicagenic acid exhibited the strongest inhibitory activity toward hyaluronidase, suggesting that this triterpenoid may contribute substantially to the observed enzyme‐binding interactions.

In summary, these UF‐HPLC and IC_50_ data demonstrated that medicagenic acid and rutin act as multi‐target ligands against PL, α‐glucosidase, and hyaluronidase. Reduced chromatographic peak intensity reflects ligand‐enzyme binding interactions rather than the direct measurement of enzyme inhibition. Although UF‐HPLC screening can effectively identify ligands that interact with enzyme targets, it should be noted that, although the UF‐HPLC screening approach can identify compounds that bind to the enzyme, it cannot directly confirm the mechanism of inhibition. Therefore, the observed reduction in peaks reflects ligand–enzyme interactions rather than verified catalytic inhibition. Further enzyme kinetic studies are required to determine the mode of inhibition (such as competitive, non‐competitive, or mixed).

### 3.4 Structural Identification of Compounds

**FIGURE 4:**
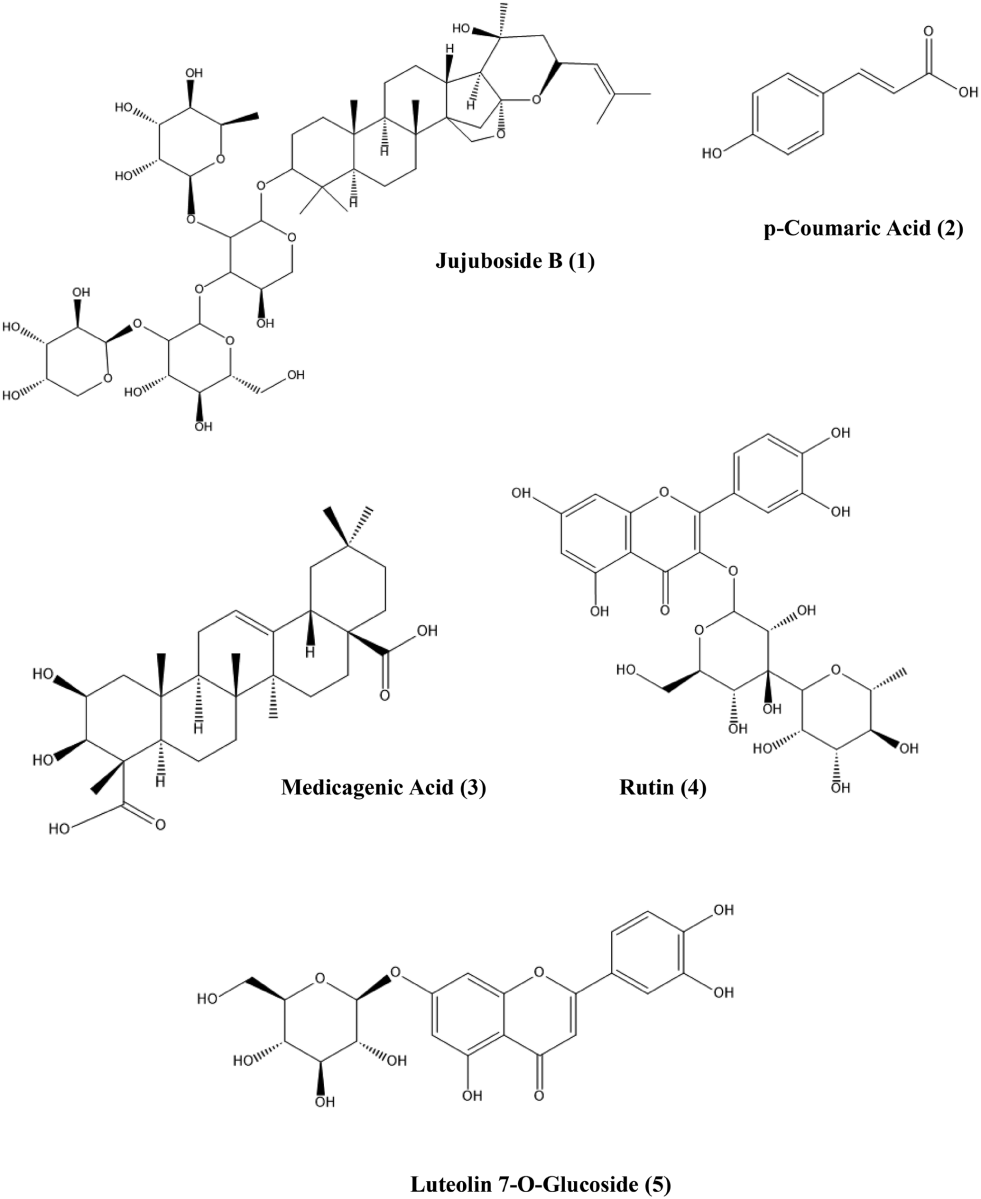
Structures of major compounds in *Z. jujuba* extract.

Jujuboside B (1) is a white amorphous powder with a m.p. of 228°C–231°C UV spectrophotometric analysis revealed only a weak triterpenoid band (204 nm) and no additional shoulder peaks, suggesting the absence of conjugated impurities. The EI‐MS (Table S4, Figure S1, Figure 4) extracted ion chromatogram (XIC 1044–1045 Da) showed a single dominant peak at m/z ≈1043 [M − H]^−^, consistent with the calculated mass for C_52_H₈_4_O₂₁ and the value for jujuboside B derived from jujube. Complementary ^1^H‐NMR data (600 MHz, DMSO‐d₆): δ 5.35 (1H, br s, H‐12), δ 4.82 (1H, d, J = 7.8 Hz, H‐1″, Glc), 4.68 (1H, d, J = 7.5 Hz, H‐1‴, xyl), 4.62 (1H, d, J = 7.6 Hz, H‐1⁗, Fuc), an overlapping sugar proton region at δ 3.90–3.20, and several tertiary methyl singulets between δ 1.08–0.78 were observed (Figure S2), confirming the presence of a triterpenoid aglycone with three sugar residues, consistent with literature data for jujuboside B (Wu et al. 2013).

p‐Coumaric acid (2) is a white amorphous powder with a m.p. of 210°C–213°C. The diode array UV spectrum showed typical hydroxycinnamic acid absorption characteristics (254 nm) with a strong maximum absorbance in the near‐ultraviolet region, closely resembling that of the p‐coumaric acid reference standard. In EI‐MS (Figure 4, Table S4, Figure S1) analysis, the XIC at 163–164 Da showed an isolated chromatographic peak for the deprotonated ion m/z ≈163 [M − H]^−^, consistent with the expected mass of C₉H₈O₃. The ^1^H NMR spectrum (600 MHz, DMSO‐d₆) showed an AA'BB′ aromatic pattern at δ 7.51 (2H, d, J = 8.6 Hz, H‐2,6) and 6.78 (2H, d, J = 8.6 Hz, H‐3,5), with trans‐configurations at δ 7.32 (1H, d, J = 15.9 Hz, H‐β) and 6.21 (1H, d, J = 15.9 Hz, H‐α), indicating the trans configuration (Figure S2). This was fully consistent with the para‐substituted trans‐cinnamic acid, confirming its assignment as p‐coumaric acid (Karthikeyan et al. 2015; Saad et al. 2019).

Rutin (4) is a yellow amorphous powder, m.p. 240°C–242°C. The UV spectrum exhibited a typical quercetin glycoside pattern along with two strong bands characteristic of flavanol O‐glycosides (254 nm). In the EI‐MS (Table S4, Figure S1, Figure 4) XIC within the 609–610 Da range, a single peak which was assigned to the deprotonated molecular ion at m/z ≈609 [M − H]^−^, and closely matched the calculated mass of C_27_H_30_O_16_ and previously reported rutin data from jujube (Bature et al. 2024). The ^1^H NMR spectrum (Figure S2) (600 MHz, DMSO‐d₆/CD₃OD) showed aromatic proton signals at δ 7.78 (1H, d, J = 9.0 Hz, H‐2″), 7.53 (1H, dd, J = 2.0, 8.5 Hz, H‐6′), 6.95 (1H, d, J = 8.5 Hz, H‐5′), 6.39 (1H, d, J = 1.5 Hz, H‐8), 6.21 (1H, d, J = 2.0 Hz, H‐6), along with anomeric signals at δ 5.47 (1H, s, H‐1″) and 4.33 (1H, br s, H‐1‴), a series of sugar multiplets between δ 3.60–3.50 and 3.57, and a terminal methyl signal at δ 1.22 (3H, br s, H‐6‴) (Figure S2), confirming that rutin (Bature et al. 2024).

Luteolin 7‐O‐glucoside (5) is a yellow powder, and m.p. 256°C–258°C. The UV spectra showed flavanone features that showed the maximum corresponding to Band II and Band I (350–370 nm). Typical of luteolin derivatives and were nearly consistent with the luteolin 7‐O‐glucoside spectra reported in dates. EI MS (Table S4, Figure 4, Figure S1) analysis with XIC (447–448 Da) showed a peak corresponding to a deprotonated ion of m/z ≈447 [M − H]^−^, which was consistent with the theoretical mass of C_21_H_20_O_11_. ^1^H NMR spectra (Figure S2) (600 MHz, DMSO‐d₆) showed flavanone proton patterns at δ 6.73 (1H, s, H‐3), 6.43 (1H, d, J = 2.0 Hz, H‐6), 6.78 (1H, d, J = 2.0 Hz, H‐8), and δ 6.89 (1H, d, J = 8.5 Hz, H‐5′), 7.40 (1H, d, J = 2.0 Hz, H‐2′), and 7.43 (1H, dd, J = 8.5, 2.0 Hz, H‐6′), δ showed a sugar multiplet corresponding to H‐2″–H‐6″ between the anomeric doublet and δ 3.9–3.1 at 5.05 (1H, d, J = 7.5 Hz, H‐1″) (Figure S2), confirming that it is luteolin 7‐O‐β‐glucoside (Lin et al. 2015).

Medicagenic acid (3) is a white amorphous powder, and m.p. 380°C–382°C. Its UV spectrum showed only very weak absorption (254 nm), as expected for a pentacyclic triterpene acid lacking extended conjugation. EI MS (Figure 4, Table S4, Figure S1) monitoring with an XIC at 501–502 Da gave one major peak corresponding to a deprotonated ion at m/z ≈501 [M − H]^−^, in agreement with the theoretical mass for C_30_H_46_O_6_. The ^1^H NMR spectrum (600 MHz, DMSO‐d₆) showed a single olefinic/oxymethine signal at δ 5.4–5.2 (1H, m, H‐12), a series of oxymethine/oxymethylene protons at δ 3.8–3.3 (H‐2, H‐3, H‐23), a broad envelope of methylene and methine ring protons between δ 2.2 and 1.2, and three tertiary methyl singlets at δ 1.10,0.90, and 0.87 (3H each) (Figure S2), which together describe a medicagenic‐acid type triterpene aglycone consistent with literature reports (Bharati et al. 2025).

### 3.5 Bioactivity Interrelationships Revealed by Correlation and Chord Diagram Analysis

**FIGURE 5:**
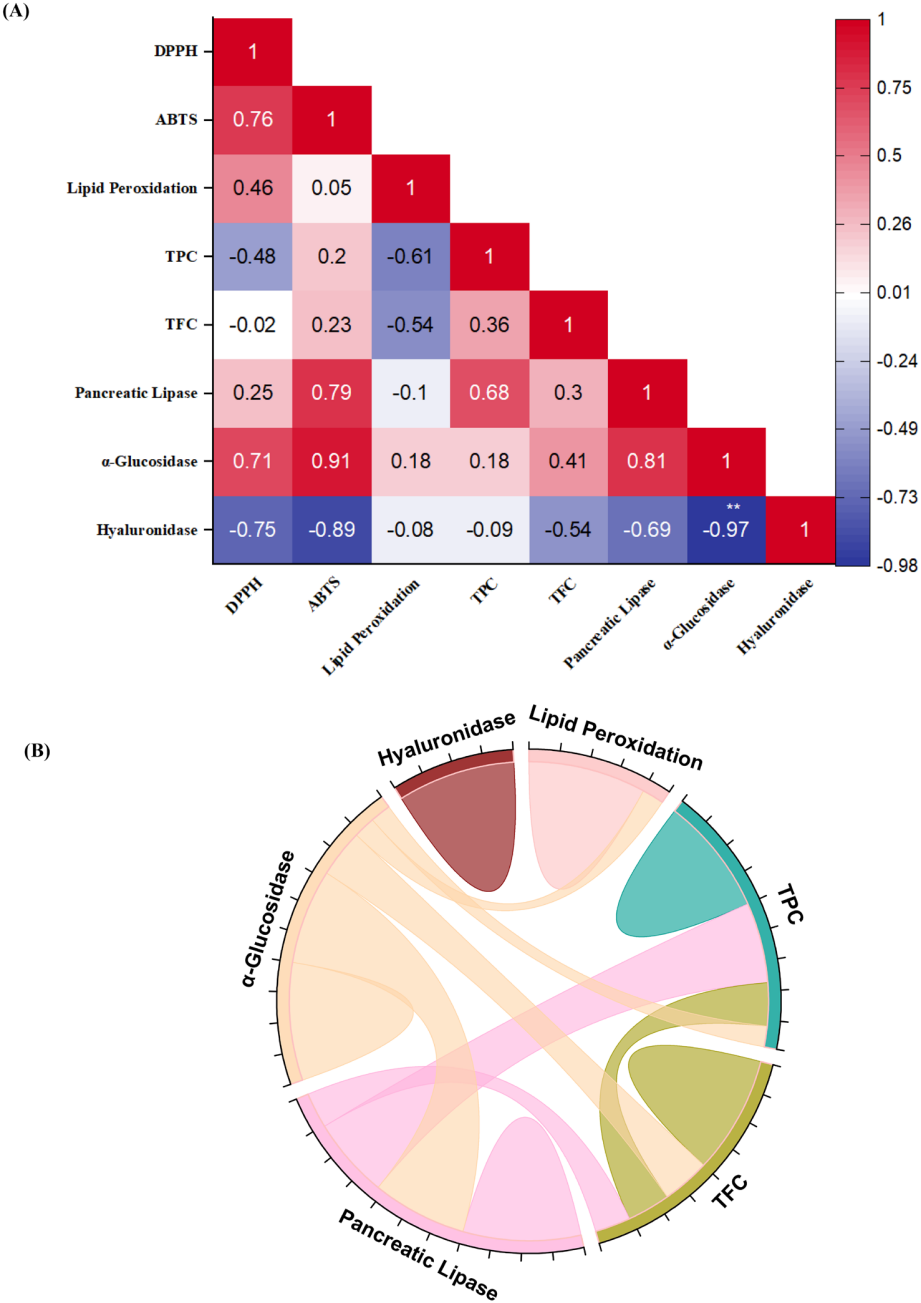
Correlation coefficient from *Z. jujuba* (A) Heat‐map for Pearson's correlation coefficients and (B) Chord diagram between bioavailability parameters (TPC mg GAE/g extract, TFC mg QE/g extract, lipid peroxidation, α‐glucosidase, PL, and hyaluronidase inhibition activity). *p* < 0.01 indicates statistical significance: A chord diagram illustrating the relationships between antioxidant capacity and enzyme inhibitory activities overall.

The relationship (Figure 5A,B) between the TPC and TFC of *Z. jujuba* extract and its biological activities. Pearson correlation analysis was performed to evaluate the statistical relationship between the measured parameters, and correlation coefficients were considered significant at *p* < 0.05. The heatmap indicates that PL inhibitory activity showed a positive correlation with both TPC (*r* ≈ 0.68) and TFC (*r* ≈ 0.30), and α‐glucosidase inhibitory activity showed a strong positive correlation with PL inhibitory activity (*r* ≈ 0.81) and a moderate positive correlation with TFC (*r* ≈ 0.41). This suggests that the flavonoid‐rich sample plays a key role in regulating carbohydrate and lipid digestive enzymes, consistent with previous reports linking *Z. jujube* phenolic compounds and flavonoids to α‐glucosidase and lipase inhibition (Bukhari et al. 2022). In contrast, lipid peroxidation inhibition showed a negative correlation with TPC and total TFC (*r* ≈ −0.61 and −0.54, respectively). This inverse relationship suggests that higher levels of phenolic and flavonoid compounds are associated with reduced oxidative lipid damage. Phenolic compounds can donate hydrogen atoms or electrons to neutralize reactive oxygen species and interrupt lipid peroxidation chain reactions. This protects biological membranes from oxidative degradation (Rice‐Evans et al. 1996). This pattern aligns with research findings that triterpenic acids in jujube, alongside phenolic compounds, exhibit potent ROS scavenging and lipid peroxidation inhibitory effects (Fu et al. 2021). A strong negative correlation was observed between α‐glucosidase and hyaluronidase inhibition (*r* = −0.97, *p* < 0.01). This suggests that potent α‐glucosidase inhibitors may not necessarily also inhibit hyaluronidase. This inverse relationship may reflect differences in enzyme binding preferences or structural requirements for inhibition. The chord diagram (Figure 5B) visualizes that hyaluronidase inhibition appeared relatively independent from the antioxidant parameters within this dataset with no connections to the antioxidant parameters, and its inhibition is just affected by specific target constituents in the *Z. jujuba* extract. This pattern suggests that flavonoid‐rich fractions may contribute to the observed enzyme inhibition activities.

### 3.6 Molecular Docking Validation

**FIGURE 6:**
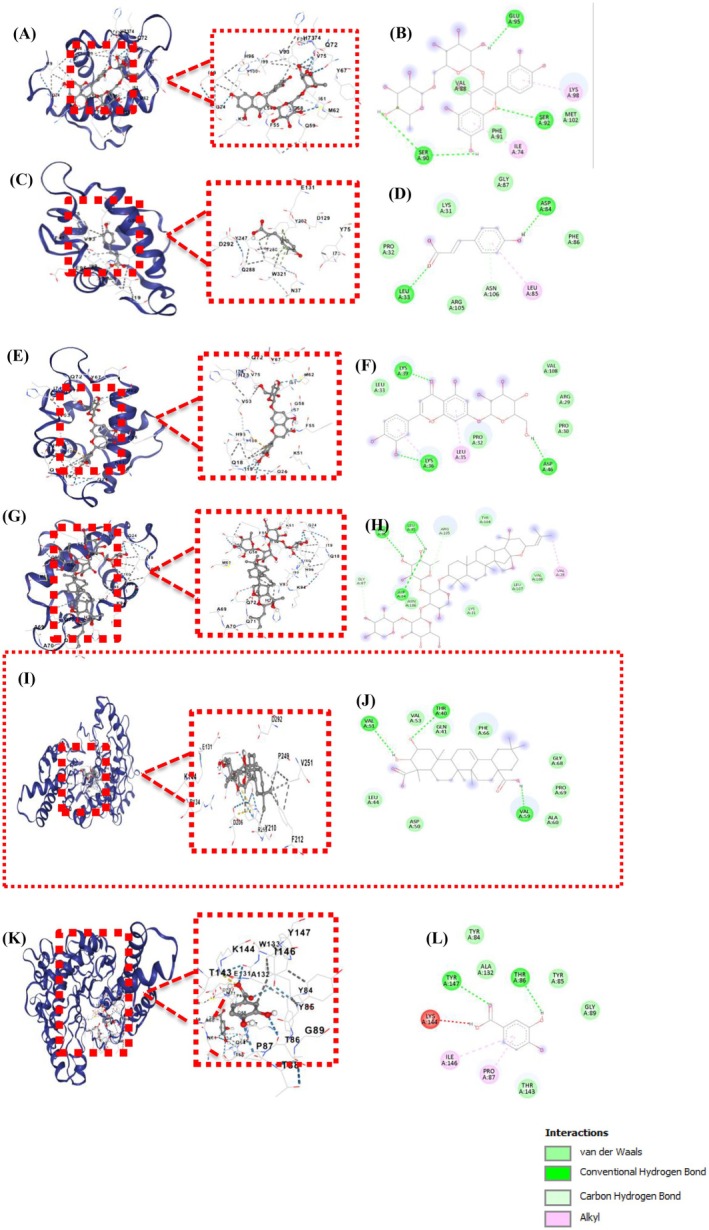
*Z. jujuba* compounds molecular docking (A) docking pose of the rutin compound among hyaluronidase. (B) interactions of rutin and hyaluronidase. (C) docking pose of p‐coumaric acid among hyaluronidase. (D) interactions of p‐coumaric acid and hyaluronidase. (E) docking pose of luteolin 7‐O‐glucoside among hyaluronidase. (F) interactions of luteolin 7‐O‐glucoside and hyaluronidase. (G) docking pose of jujuboside B among hyaluronidase. (H) interactions of jujuboside B and hyaluronidase. (I) docking pose of medicagenic acid among hyaluronidase. (J) interactions of medicagenic acid and hyaluronidase, and (K) docking pose of tannic acid among hyaluronidase. (L) interactions of tannic acid and hyaluronidase. Dashed lines indicate the constructed bonds between the ligand and enzyme. Green dashed lines indicate the hydrogen bonds.

Molecular docking analyses (Figure 6A–J) provided mechanistic insight into the binding interactions underpinning the hyaluronidase inhibitory activities observed in *Z. jujuba* extracts. The study focused on predominant bioactive compounds identified by isolation screening, including jujuboside B (1), p‐coumaric acid (2), rutin (4), luteolin 7‐O‐glucoside (5), and medicagenic acid (3), which is newly characterized. All selected molecules were docked against the hyaluronidase active site using contemporary, validated computational protocols that ensure representative ligand energetics and poses (Abdulqahar et al. 2023; Mahdi et al. 2024). To validate the docking results in context, tannic acid, a reported hyaluronidase inhibitor, was docked under identical conditions and used as a reference ligand to compare binding affinities.

Table 2 summarizes the binding affinities and key amino acid interactions for major *Z. jujuba* bioactive docked against the hyaluronidase enzyme. Among the compounds, medicagenic acid showed the most favorable docking score (−7.4 kcal/mol) with hydrogen bonds to catalytic residues THR40, GLN41, and ASP50, consistent with its UF‐HPLC binding (36.39% peak reduction in Table 1). Jujuboside B exhibited a binding energy of −6.4 kcal/mol with notable interactions involving ASP84, PHE86, GLY87, and LEU85, while luteolin 7‐O‐glucoside showed a binding energy of −5.5 kcal/mol, forming contacts with LYS39, LYS36, LEU35, ASP46, and PRO32. Rutin and p‐coumaric acid had binding energies of −5.2 and −4.0 kcal/mol, respectively, interacting with GLU95, SER90, SER92, VAL88 (rutin), and ASP84, ASN106, LEUA33 (p‐coumaric acid). These data further highlight the diversity and specificity in the residue‐level interactions underpinning the inhibitory potential of these natural ligands (Gunasekaran et al. 2023). For comparison, the reference inhibitor tannic acid had a docking energy of −6.7 kcal/mol, and it also demonstrated a strong interaction with the hyaluronidase active pocket. As illustrated in (Figure 6K,L). Tannic acid formed multiple hydrogen bonds with key residues, such as GLN‐41, ASP‐50, and PHE‐66. Additional stabilization was provided by extensive van der Waals interactions with surrounding residues, including VAL‐51, LEU‐44, and ALA‐60. These interactions suggest that tannic acid can occupy the catalytic region of the enzyme and form a stable ligand enzyme complex.

**TABLE 2:** Amino acid residues on the *Z. jujuba* compounds that interact with the hyaluronidase and ligands.

Ligand	Binding energy (Vina score, kcal/mol)	Key interactions	Interaction type	Distance (Å)
Rutin	−5.2	GLU95, SER90, SER92, VAL88	Hydrogen bond	2.9
p‐Coumaric acid	−4.0	ASP84, ASN106, LEUA33	Hydrogen bond	2.7
Luteolin 7‐O‐Glucoside	−5.5	LYS39, LYS36, LEU35, ASP46, PRO32	Hydrophobic interaction	3.8
Jujuboside B	−6.4	ASP84, PHE86, GLY87, and LEU85	Hydrophobic interaction	3.9
Medicagenic acid	−7.4	THR40, GLN41, ASP50, VAL‐51, THR‐40, GLN‐41, PHE‐66, LEU‐44, ASP‐50, ALA‐60	Hydrogen bond, van der Waals	2.8
Tannic acid	−6.7	GLN‐41, ASP‐50 PHE‐66, VAL‐51, LEU‐44, ALA‐60	Hydrogen bond, van der Waals	2.83

Medicagenic acid, as a novel hyaluronidase interactor from *Z. jujuba* , has significant affinity for the active pocket of hyaluronidase (Figure 6I,J). Medicagenic acid establishes multiple hydrogen bonds, especially with key catalytic residues such as VAL‐51, THR‐40, GLN‐41, and PHE‐66, as well as extensive van der Waals interactions with LEU‐44, ASP‐50, and ALA‐60. These interaction networks suggest a stabilizing role for medicagenic acid, and the presence of both hydrophilic and hydrophobic contacts implies a strong fit within the enzyme's binding groove, consistent with the optimal pharmacophore requirements for hyaluronidase peak area reduction recently described in high‐impact mechanistic studies (Mahdi et al. 2024; Hertel et al. 2006; Mohamed et al. 2020). Medicagenic acid score (−7.4 kcal/mol) is comparable to that of several previously reported natural hyaluronidase inhibitors. For example, it showed a binding energy of approximately Narcissin −6.853, quercetin‐3‐O‐glucoside −6.088, hydroxyanigorufone −4.306, (Mohamed et al. 2020) rosmarinic acid, −8.2 kcal/mol, while vanillic acid and luteolin 7‐o‐glucuronide derivatives exhibited docking energies between −5.7 and −9.6 kcal/mol (Karatoprak et al. 2022). By contrast, tannic acid showed a docking energy of −6.7 kcal/mol in the present study, whereas stronger binding affinities of approximately −9.4 kcal/mol have been reported for polyphenolic inhibitors such as tannic acid in previous studies (Karatoprak et al. 2022).

The interaction of medicagenic acid, also compared with flavonoids such as rutin, may be related to its triterpenoid structure, which provides a hydrophobic backbone that can form stable van der Waals interactions within the enzyme binding pocket. Meanwhile, its polar functional groups enable hydrogen bonding. Similar structure–activity relationships have been reported for other plant‐derived triterpenoids exhibiting hyaluronidase inhibitory activity (Lee et al. 2008; Tan et al. 2015; Yang et al. 2024).

In summary, medicagenic acid broadened the structural diversity of active inhibitors by engaging both polar and nonpolar residues, potentially enhancing the overall enzyme‐inhibitory efficacy of the phytochemical mixture. Energetic analyses showed that medicagenic acid ranked among the highest for docking scores, in line with its strong in vitro effect and reinforcing the validity of the in silico model (Lu et al. 2025; Thoyajakshi et al. 2024; Rungruang et al. 2025). This concordance between docking scores, UF‐HPLC peak‐area depletion and in vitro IC_50_ data suggests that medicagenic acid may represent an important contributor to the hyaluronidase inhibitory activity, and rutin, jujuboside B, and luteolin 7‐O‐glucoside also acting as important co‐contributors. Comparing the results with those of the reference inhibitor further supports the idea that the predicted binding energies of several *Z. jujuba* constituents fall within the range typically reported for natural hyaluronidase inhibitors. This strengthens the reliability of the docking interpretation (Zhou 2025; Syamsul et al. 2022).

Although docking provides valuable insights into potential ligand‐enzyme interactions, further experimental validation, such as enzyme kinetic analysis or cellular assays, is required to confirm the exact mechanism of enzyme binding (Memarpoor‐Yazdi et al. 2020).

### 3.7 Limitations and Future Directions

This study focuses on in vitro enzyme inhibition assays and computational docking analysis. While these approaches offer valuable insights into potential enzyme ligand interactions, they do not accurately reflect biological conditions. Cellular validation, bioavailability assessment, and skin permeation studies are required before any translational or therapeutic claims can be made. Therefore, future research should evaluate the cytotoxicity and efficacy of the identified compounds in human dermal fibroblast models, as well as their formulation stability and potential in vivo anti‐aging effects, to confirm their biological relevance.

## 4 Conclusion

This study systematically elucidates the antioxidant and enzyme inhibitory landscape of South Korean *Z. jujuba* , integrating in vitro bioassays, chromatographic, correlation analysis, and molecular docking approaches to associate bioactivity profiles with specific phytochemical constituents. The multi‐target inhibition (especially, hyaluronidase inhibition) supports *Z. jujuba's* versatility as a therapeutic candidate for skin aging and oxidative stress. One of the identified constituents, medicagenic acid, exhibited the strongest interaction with hyaluronidase, suggesting that it may significantly contribute to the observed inhibitory activity in the extract. Combined UF‐HPLC screening, IC_50_ evaluation, and molecular docking analysis provide preliminary insights into the mechanisms of enzyme–ligand interaction within *Z. jujuba*. Overall, these insights enhance our scientific understanding of the bioactive composition of *Z. jujuba* and its potential relevance to dermatological and functional food research. However, further studies are necessary to fully confirm the inhibitory mechanisms and biological relevance of these compounds, including enzyme kinetic analysis, cellular validation, and bioavailability evaluation.

## Author Contributions

**Sichan Kim:** methodology. **Soon Sung Lim:** writing – review and editing, funding acquisition, supervision. **Zhaoyang Wu:** methodology. **Soo Kyeong Lee:** methodology. **Saba Noreen:** conceptualization, methodology, validation, formal analysis, resources, data curation, writing – original draft, visualization. **Deokho Lee:** formal analysis, investigation, data curation, writing – original draft, writing – review and editing, supervision, project administration. **Ji Won Choi:** methodology.

## Funding

This research was supported by Hallym University Research Fund, 2023 (HRF‐202304‐007).

## Ethics Statement

The authors have nothing to report.

## Consent

The authors have nothing to report.

## Conflicts of Interest

The authors declare no conflicts of interest.

## Supporting information


**Figure S1:** EI‐MS (m/z) data of isolated compounds: jujuboside B, p‐coumaric acid, medicagenic acid, rutin, and Luteolin‐7‐O‐Glucoside.
**Figure S2:** NMR data of isolated compounds: jujuboside B, p‐coumaric acid, medicagenic acid, rutin, and Luteolin‐7‐O‐Glucoside.
**Table S1:** HPLC gradient program.
**Table S2:** Comparison of natural hyaluronidase inhibitors reported in the literature with medicagenic acid.
**Table S3:** Raw hyaluronidase inhibition data showing individual replicate values used for IC_50_ determination. Data obtained from two independent experimental replicates. The mean inhibition percentages were used for IC_50_ calculation.
**Table S4:** EI‐MS data (m/z) for compounds isolated from *Z. jujuba*.

## Data Availability

The datasets generated and analyzed during the current study are available from the corresponding author upon reasonable request.
