# Correction to “Correlational and Molecular Mechanism Analyses of Bioactive Compounds From *Ziziphus jujuba*: Focusing on Anti‐Hyaluronidase and Antioxidant Networks”

**DOI:** 10.1002/fsn3.71974

**Published:** 2026-05-27

Noreen S, Wu Z, Kim S, Choi JW, Lee SK, Lee D, Lim SS. Correlational and Molecular Mechanism Analyses of Bioactive Compounds From *Ziziphus jujuba*: Focusing on Anti‐Hyaluronidase and Antioxidant Networks. *Food Science and Nutrition* 2026;14(5): e71844. https://doi.org/10.1002/fsn3.71844.

In the Funding section, a funding source was omitted. The statement should read as follows:

“This research was supported by Hallym University Research Fund, 2023 (HRF‐202304‐007). Furthermore, the current study was supported by the Basic Science Research Program through the National Research Foundation of Korea (NRF), funded by the Ministry of Education (Grant: 2340056192; NR060133).”

We apologize for this error.

