# Supplementary material for: Correlational and Molecular Mechanism Analyses of Bioactive Compounds From Ziziphus jujuba: Focusing on Anti‐Hyaluronidase and Antioxidant Networks
Source: Food Sci Nutr. 2026 May 7;14(5):e71844. doi: 10.1002/fsn3.71844 (PMC13151021; doi:10.1002/fsn3.71844)
Supplement: Supplementary file 1 — Figure S1: EI‐MS (m/z) data of isolated compounds: jujuboside B, p‐coumaric acid, medicagenic acid, rutin, and Luteolin‐7‐O‐Glucoside. Figure S2: NMR data of isolated compounds: jujuboside B, p‐coumaric acid, medicagenic acid, rutin, and Luteolin‐7‐O‐Glucoside. Table S1: HPLC gradient program. Table S2: Comparison of natural hyaluronidase inhibitors reported in the literature with medicagenic acid. Table S3: Raw hyaluronidase inhibition data showing individual replicate values used for IC50 determination. Data obtained from two independent experimental replicates. The mean inhibition percentages were used for IC50 calculation. Table S4: EI‐MS data (m/z) for compounds isolated from Z. jujuba. [file FSN3-14-e71844-s001.docx]

[M+Na]^+^

[M+K]^+^

Other fragment ions are potassium cluster ions

**Jujuboside B
MW = 1045**

**p-coumaric acid
MW =164**

[2M-H]^+^

[M-H-H2O]^+^

[M-H-CO_2_]^+^

[M-H]^+^

[2M-H+Na]^+^

[2M-H]^+^

[M-H]^+^

**Medicagenic acid
MW =502**

[2M-H+Na]^+^

[2M-H]^+^

[2M-H+2]^+^

[M-H+Na]^+^

[M-H+2]^+^

[M-H]^+^

**Rutin
MW =610**

[M-H]^+^

[2M-H+Na]^+^

[2M-H]^+^

**Luteolin‑7‑O‑glucoside
MW =448**

**Supplementary Fig. 1.** EI-MS (m/z) data of isolated compounds: jujuboside B, p-coumaric acid, medicagenic acid, rutin, and Luteolin‑7‑O-Glucoside.


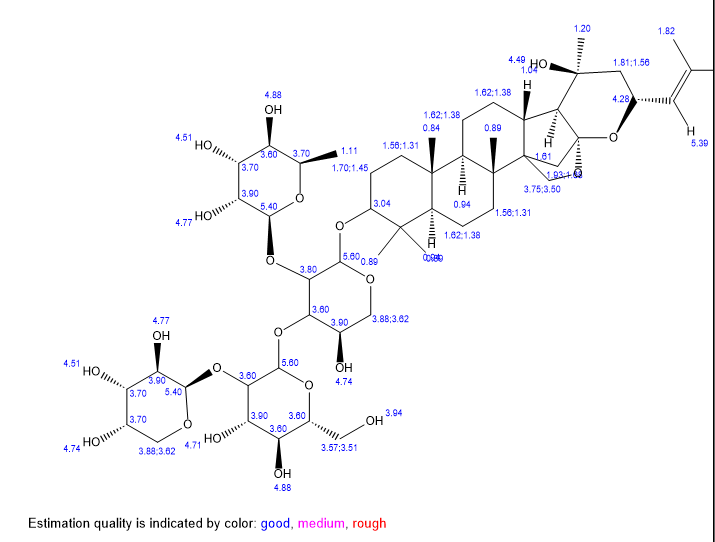


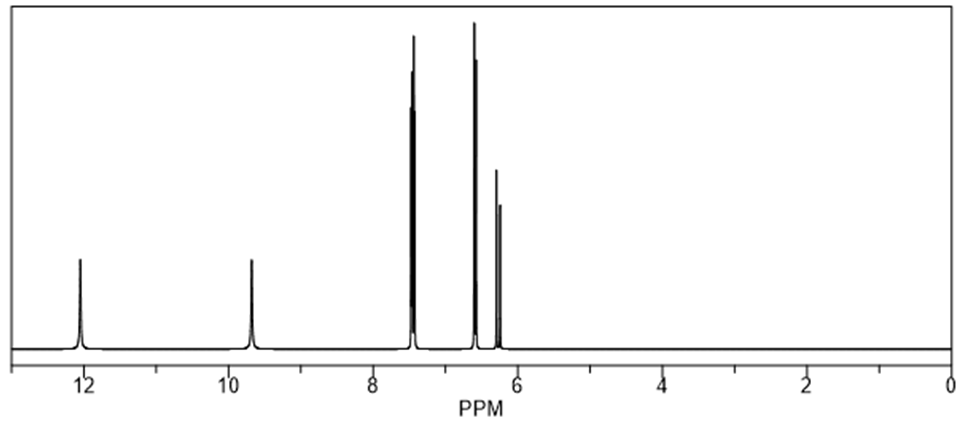

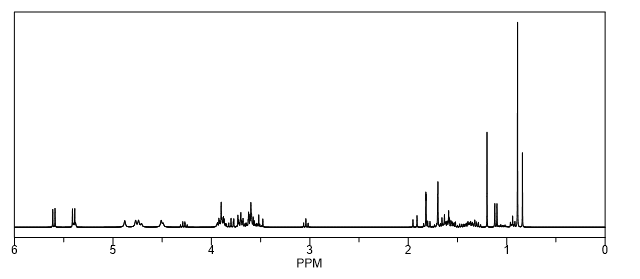

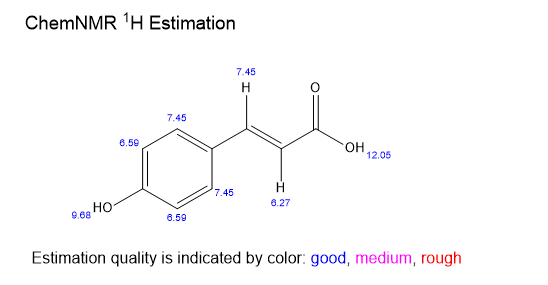

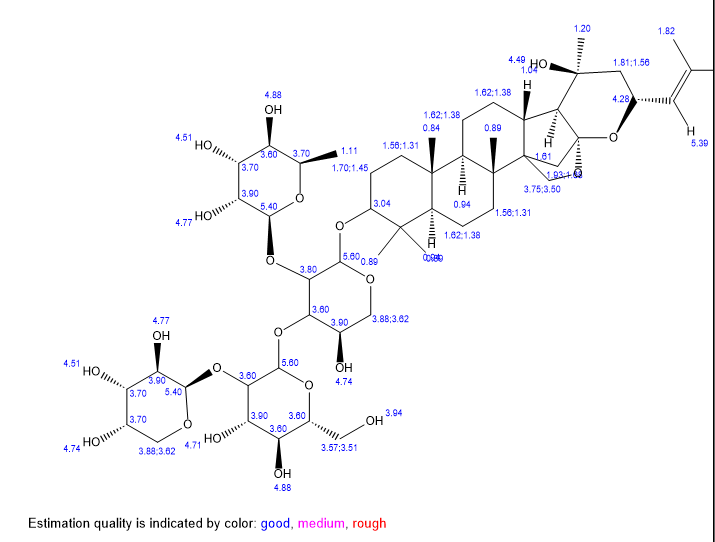


**p-coumaric acid**

Jujuboside B

p-coumaric acid


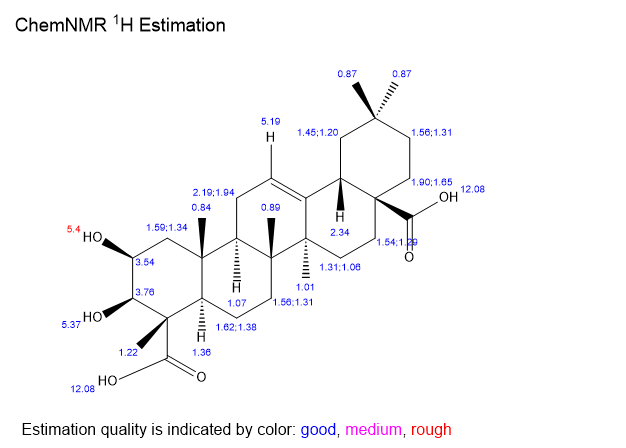

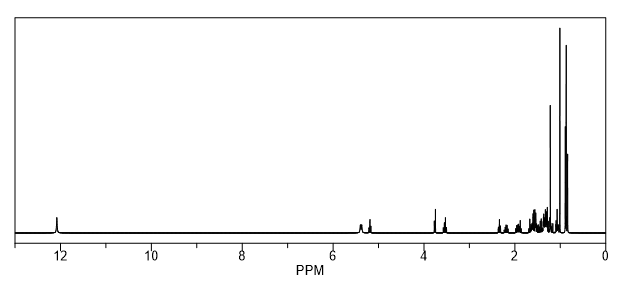


Medicagenic acid


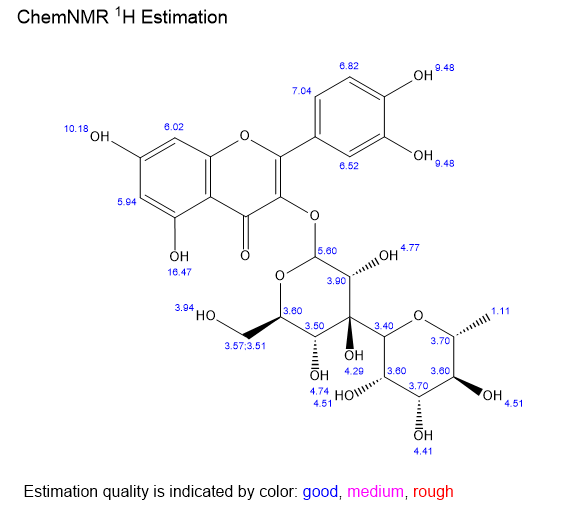

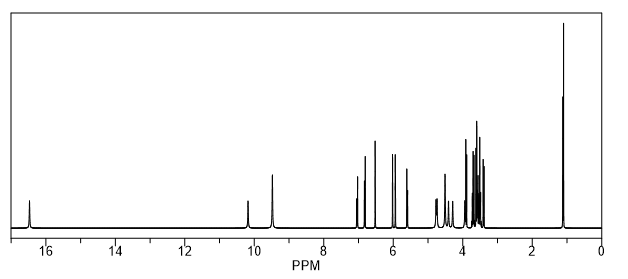


Rutin


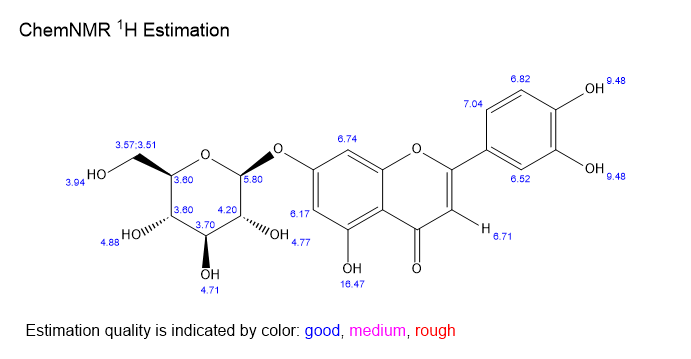

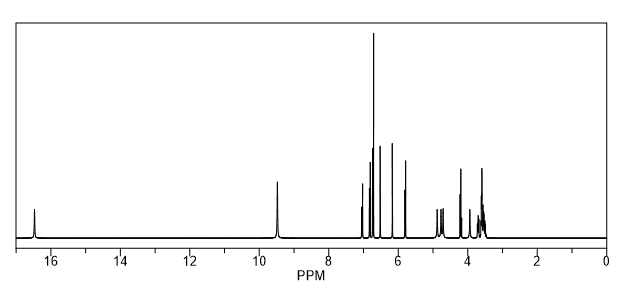


luteolin‑7‑O‑glucoside

**Supplementary Fig. 2.** NMR data of isolated compounds: jujuboside B, p-coumaric acid, medicagenic acid, rutin, and Luteolin‑7‑O-Glucoside.

**Supplementary Table 1.** HPLC gradient program.

| **Time** | **Solvent A (%), 0.1% formic acid** | **Solvent B (%), Acetonitrile** |
| --- | --- | --- |
| 0 | 90 | 10 |
| 5 | 90 | 10 |
| 40 | 80 | 20 |
| 55 | 0 | 100 |
| 60 | 0 | 100 |
| 65 | 90 | 10 |
| 75 | 90 | 10 |

**Supplementary Table 2.** Comparison of natural hyaluronidase inhibitors reported in the literature with medicagenic acid.

| **Compound** | **IC_50_** | **Reference** |
| --- | --- | --- |
| Medicagenic Acid | 113 µM | - |
| Glycyrrhizin | 3 µM | [1] |
| Rosmarinic acid | 240 µM | [2] |
| Chicoric acid | 171 μM | [3] |
| Nuciferine | 240 µM | [4] |

**Supplementary Table 3**. Raw hyaluronidase inhibition data showing individual replicate values used for IC₅₀ determination. Data obtained from two independent experimental replicates. The mean inhibition percentages were used for IC₅₀ calculation.

| Compounds | Sample Concentration (ug/mL) | Mean ±SD |
| --- | --- | --- |
| *Z. jujuba* | 100 | 62.60 ± 2.91 |
|  | 50 | 29.59 ± 0.39 |
|  | 10 | 17.81 ± 0.77 |
| Jujuboside B | 100 | 78.32 ± 0.99 |
|  | 50 | 54.55 ± 0.99 |
|  | 10 | 23.08 ± 0 |
| Rutin | 100 | 83.92 ± 0.99 |
|  | 50 | 41.96 ± 0.99 |
|  | 10 | 23.08 ± 0 |
| Medicagenic acid | 100 | 94.41 ± 1.98 |
|  | 50 | 53.15 ± 2.97 |
|  | 10 | 32.17 ± 2.97 |
| p-Coumaric acid | 100 | 60.14 ± 0.99 |
|  | 50 | 39.16 ± 0.99 |
|  | 10 | 14.69 ± 1.98 |
| Luteolin-7-O-glucoside | 100 | 75.52 ± 0.99 |
|  | 50 | 51.05 ± 1.98 |
|  | 10 | 26.57 ± 0.99 |
| Tannic acid | 50 | 75.52 ± 0.42 |
|  | 10 | 53.39 ± 1.67 |
|  | 2 | 18.44 ± 1.46 |

**Supplementary Table 4.** EI-MS data (m/z) for compounds isolated from *Z. jujuba.*

| **Peak no** | **Compound** | **Formula** | **Exact mass** | **M-H^-^** |
| --- | --- | --- | --- | --- |
| 1 | Jujuboside B | C_52_H_84_O_21_ | 1044.55 | 1043 |
| 2 | P-Coumaric Acid | C_9_H_8_O_3_ | 164.2 | 163 |
| 3 | Medicagenic Acid | C_30_H_46_O_6_ | 502.33 | 501 |
| 4 | Rutin | C_27_H_30_O_16_ | 610.15 | 609 |
| 5 | Luteolin 7-O-Glucoside | C_21_H_20_O_11_ | 448.10 | 447.09 |

**References**

1. Furuya, T., et al., *Biochemical characterization of glycyrrhizin as an effective inhibitor for hyaluronidases from bovine testis.* Biological and Pharmaceutical Bulletin, 1997. **20**(9): p. 973-977.

2. Myose, M., T. Warashina, and T. Miyase, *Triterpene saponins with hyaluronidase inhibitory activity from the seeds of Camellia sinensis.* Chemical and Pharmaceutical Bulletin, 2012. **60**(5): p. 612-623.

3. Lengers, I., et al., *Improved surface display of human Hyal1 and identification of testosterone propionate and chicoric acid as new inhibitors.* Pharmaceuticals, 2020. **13**(4): p. 54.

4. Morikawa, T., et al., *Quantitative determination of principal aporphine and benzylisoquinoline alkaloids due to blooming state in lotus flower (flower buds of Nelumbo nucifera) and their hyaluronidase inhibitory activity.* Natural Product Communications, 2019. **14**(6): p. 1934578X19857834.
